# Factors influencing the outcomes of dermatoses during the COVID-19 outbreak in China: a retrospective study

**DOI:** 10.3389/fmed.2024.1417358

**Published:** 2024-05-30

**Authors:** Jing-Hui Li, Si-Zhe Li, Si-Hang Wang, Jie Zhang, Ying-Han Xie, Ya-Gang Zuo

**Affiliations:** ^1^Department of Dermatology, State Key Laboratory of Complex Severe and Rare Diseases, National Clinical Research Center for Dermatologic and Immunologic Diseases, Peking Union Medical College Hospital, Chinese Academy of Medical Sciences and Peking Union Medical College, Beijing, China; ^2^School of Medicine, Tsinghua University, Beijing, China

**Keywords:** COVID-19, biologics, vitiligo, psoriasis, autoimmune bullous disease

## Abstract

**Background:**

The coronavirus disease 2019 (COVID-19) pandemic subverted people’s lives and potentially affected the management and prognosis of pre-existing dermatoses. The study aims to identify factors influencing the outcomes of dermatoses during a rapid and widespread Omicron outbreak in China following the adjustment of the COVID-19 policy.

**Materials and methods:**

This retrospective observational study involved outpatients visiting the dermatology department at a tertiary referral hospital in Beijing, China between December 2022 and February 2023. Demographics, COVID-19 characteristics, treatment modalities, and dermatosis outcomes were subjected to statistical analysis.

**Results:**

The odds ratio (OR) for vitiligo aggravation during COVID-19 was 0.497 [95% confidence interval (CI): 0.254–0.973, *p* = 0.038] compared to total patients with various dermatoses. Psoriasis patients with a maximum body temperature (T_max_) over 38.6°C during COVID-19 were 2.833 times more likely to experience dermatosis aggravation (OR: 2.833 [1.029–7.803], *p* = 0.041). Moreover, autoimmune bullous disease (AIBD) patients receiving biologics treatment exhibited a reduced likelihood of aggravation during the COVID-19 outbreak (OR: 0 [0–0.531], *p* = 0.011).

**Conclusion:**

Vitiligo exhibits lower aggravation rates during COVID-19 than other dermatoses. A higher body temperature during COVID-19 infection can increase the risk of psoriasis aggravation. Biologics treatment reduces the risk of AIBD aggravation during the COVID-19 outbreak.

## 1 Introduction

The novel severe acute respiratory syndrome coronavirus 2 (SARS-CoV-2) is the culprit agent of the coronavirus disease 2019 (COVID-19) pandemic, and the Omicron strain currently prevails as the dominant virus type affecting people worldwide. A range of cutaneous manifestations associated with SARS-CoV-2 have been reported, with chilblain-like and maculopapular lesions being the most frequently observed (1). The pathophysiology of such lesions primarily involves the antiviral inflammatory response mounted by the immune system, rather than a direct cytopathic effect of the virus (2).

However, studies on the outcomes of various dermatoses during the COVID-19 outbreak have been limited. Here, we aimed to identify factors influencing the prognosis of pre-existing dermatoses during a rapid and widespread Omicron outbreak in China after the adjustment of the COVID-19 policy.

## 2 Materials and methods

We performed a single-center retrospective observational study on consecutive outpatients visiting the dermatology clinic at Peking Union Medical College Hospital in China from 21 December 2022 to 4 February 2023, during the first phase of the Omicron pandemic. The study was approved by the Bioethics Committee of Peking Union Medical College Hospital (No. K23C2463). Inclusion criteria were individuals (1) aged 18 years or older; (2) with a pre-existing diagnosis of eczematous disorders, psoriasis, disorders of pilosebaceous units (including acne, folliculitis, rosacea, and hidradenitis suppurativa), vitiligo, autoimmune bullous diseases (AIBD, including pemphigus, bullous pemphigoid, and mucous membrane pemphigoid), or urticaria based on guideline indicators; and (3) with a disease duration exceeding 6 months. Dermatologists provided in-person guidance on completing a paper-based questionnaire to eligible patients during their dermatology clinic appointments, following the acquisition of informed consent. Data on demographics, COVID-19 characteristics, treatments, and dermatosis outcomes were collected. COVID-19 cases were identified by positive results of SARS-CoV-2 nucleic acid or antigen tests within the past 3 months. Aggravation of the dermatosis was defined as the expansion of pre-existing skin lesions, the occurrence of new skin lesions, or the exacerbation of pruritus or pain after COVID-19 infection or within the past 3 months. Biologics treatment included Ixekizumab, Secukinumab, Ustekinumab, Dupilumab, Rituximab, and Benralizumab. *p*-values, odds ratios (OR), and 95% confidence intervals (CI) for categorical variables were calculated using Pearson’s chi-square test. *p*-values for the continuous variable (age) were calculated using Student’s t-test. A *p*-value less than 0.05 was considered statistically significant.

## 3 Results

### 3.1 Clinicodemographic profile of study participants

We contacted all patients who visited our clinic during the study period (*n* = 1,175). Among them, 568 patients consented to participate and 516 completed the questionnaire. Table 1 summarizes the clinicodemographic profile of participants with various dermatoses. Out of the 516 participants, 267 (51.7%) were female and the average age was 38.8 ± 19.1 years. Twenty-two (4.3%) patients received biologics treatment prior to the Omicron pandemic. Four hundred and thirty (83.3%) patients suffered from SARS-CoV-2 infection in the past month, among whom 396 (92.1%) developed a fever during infection, with an average maximum body temperature (T_max_) of 38.6°C ± 0.7°C and a fever duration of 2.4 ± 1.6 days. Notably, subjects with AIBD exhibited the lowest T_max_ (38.2°C ± 0.8°C), while those with disorders of pilosebaceous units showed the highest (38.8°C ± 0.7°C). The extensive use of systemic corticosteroids in patients with AIBD may partly contribute to this phenomenon.

**Table 1:** Demographic and clinical features of patients with different dermatological conditions.

	Case	Age (year)	Sex (female)	Biologics treatment	SARS-CoV-2 infection	T_max_ (°C)	Fever duration (day)
ED	202	43.2 (± 18.7)	103 (51.0%)	3 (1.5%)	166 (82.2%)	38.6 (± 0.7)	2.4 (± 1.9)
Psoriasis	91	39.2 (± 15.6)	47 (51.7%)	12 (13.2%)	77 (84.6%)	38.6 (± 0.7)	2.2 (± 1.0)
DPU	87	26.6 (± 11.1)	53 (60.9%)	0 (0%)	74 (85.1%)	38.8 (± 0.7)	2.1 (± 1.0)
Vitiligo	71	25.5 (± 16.0)	27 (38.0%)	0 (0%)	62 (87.3%)	38.6 (± 0.6)	2.6 (± 1.7)
AIBD	44	61.5 (± 15.9)	26 (59.1%)	7 (15.9%)	32 (72.7%)	38.2 (± 0.8)	2.7 (± 1.9)
Urticaria	21	41.5 (± 13.3)	11 (52.4%)	0 (0%)	19 (90.5%)	38.6 (± 0.8)	2.3 (± 0.6)
Total	516	38.8 (± 19.1)	267 (51.7%)	22 (4.3%)	430 (83.3%)	38.6 (± 0.7)	2.4 (± 1.6)

T_max_, maximum body temperature; ED, eczematous disorders; DPU, disorders of pilosebaceous units; AIBD, autoimmune bullous diseases.

### 3.2 Outcomes of pre-existing dermatoses during COVID-19 outbreak

Out of the 516 patients, 139 (26.9%) experienced aggravation of their pre-existing dermatoses, while 377 (73.1%) did not (Table 2). The number of patients with a recent SARS-CoV-2 infection is 118 (84.9%) for the aggravation group, and 312 (82.8%) for the non-aggravation group. No significant difference was detected in age, sex, biologics treatment, SARS-CoV-2 infection, and COVID-19 characteristics between the aggravation and non-aggravation groups. On univariate analysis, the OR of vitiligo aggravation was 0.497 (95% CI: 0.254–0.973, *p* = 0.038) compared to the total participants, with no significant OR detected for other dermatoses. Psoriasis patients (Table 3) with a T_max_ over 38.6°C (the average T_max_ of total participants) during COVID-19 were 2.833 times more likely to experience dermatosis aggravation (OR: 2.833 [1.029–7.803], *p* = 0.041). AIBD patients receiving biologics treatment were less likely to aggravate during the COVID-19 outbreak (OR: 0 [0–0.531], *p* = 0.011). Meanwhile, biologics treatment did not increase the risk of aggravation for psoriasis patients (OR: 0.977 [0.194–4.156], *p* = 1). Other factors, including age, sex, SARS-CoV-2 infection, fever, and fever duration, had no significant impacts on outcomes of psoriasis or AIBD. Results for other dermatoses are shown in Supplementary Table 1, with no significant risk factors detected.

**Table 2:** Comparison between patients with aggravation and non-aggravation outcomes of pre-existing dermatoses.

Characteristic		Aggravation (*n* = 139)	Non-aggravation (*n* = 377)	*p*-value	OR (95% CI)
Age, in years		40.3 (± 17.7)	38.2 (± 19.5)	0.257	
Sex (female)		79 (56.8%)	188 (49.9%)	0.160	1.324 (0.895–1.958)
Biologics treatment		4 (2.9%)	18 (4.8%)	0.344	0.591 (0.143–1.840)
SARS-CoV-2 infection		118 (84.9%)	312 (82.8%)	0.564	1.171 (0.685–2.000)
COVID-19 characteristics	Fever	110 (93.2%)	287 (92.0%)	0.668	1.198 (0.524–2.736)
	T_max_ (>38.6°C)	64 (58.2%)	145 (50.5%)	0.171	1.363 (0.874–2.124)
	Fever duration (>2 days)	87 (79.1%)	210 (73.2%)	0.224	1.387 (0.818–2.353)
Diagnosis of dermatosis	ED	57	145	0.729	1.066 (0.742–1.533)
	Psoriasis	30	61	0.237	1.334 (0.827–2.152)
	DPU	18	69	0.219	0.708 (0.407–1.231)
	Vitiligo	11	60	0.038^a^	0.497 (0.254–0.973)
	AIBD	16	28	0.180	1.550 (0.758–3.068)
	Urticaria	7	14	0.519	1.346 (0.536–3.430)

OR, odds ratio; CI, confidence interval; T_max_, maximum body temperature; ED, eczematous disorders; DPU, disorders of pilosebaceous units; AIBD, autoimmune bullous diseases. ^a^Values that are statistically significant (2-sided *p* ≤ 0.05).

**Table 3:** Comparison between patients with aggravation and non-aggravation outcomes of psoriasis and autoimmune bullous diseases.

Psoriasis				
Characteristic	Aggravation (*n* = 30)	Non-aggravation (*n* = 61)	*p*-value	OR (95% CI)
Age, in years	37.2 (± 15.4)	40.2 (± 15.8)	0.390	
Sex (female)	18	29	0.264	0.604 (0.249–1.466)
Biologics treatment	4	8	1	0.977 (0.194–4.156)
SARS-CoV-2 infection	26	51	0.704	1.275 (0.364–4.458)
Fever	25	49	0.453	1.020 (0.088–11.805)
T_max_ (>38.6°C)	17	21	0.041^a^	2.833 (1.029–7.803)
Fever duration (>2 days)	18	34	0.816	1.134 (0.392–3.286)
**AIBD**				
**Characteristic**	**Aggravation (*n* = 16)**	**Non-aggravation (*n* = 28)**	***p*-value**	**OR (95% CI)**
Age, in years	59.6 (± 13.1)	62.6 (± 17.4)	0.546	
Sex (female)	10	16	0.728	0.800 (0.227–2.817)
Biologics treatment	0	7	0.011^a^	0 (0–0.531)
SARS-CoV-2 infection	14	18	0.075	3.889 (0.731–20.682)
Fever	10	13	0.306	0.962 (0.204–4.539)
T_max_ (>38.6°C)	3	3	0.340	1.429 (0.220–9.262)
Fever duration (>2 days)	8	10	0.383	1.200 (0.160–9.013)

OR, odds ratio; CI, confidence interval; T_max_, maximum body temperature; AIBD, autoimmune bullous diseases. ^a^Values that are statistically significant (2-sided *p* ≤ 0.05).

## 4 Discussion

This study was initiated during a critical period when Omicron was rapidly spreading across China. Stringent COVID-19 regulations have been implemented in China since early 2020, effectively preventing widespread infection among the population. Following the relaxation of these restrictions in December 2022, a significant number of people contracted their first SARS-CoV-2 infection within a relatively short timeframe.

SARS-CoV-2 was known to invade pulmonary cells through angiotensin-converting enzyme 2 (ACE2) receptors. While ACE2 receptors predominantly expressed on pulmonary cells, their nonnegligible expression in epidermal keratinocytes could account for various cutaneous manifestations associated with COVID-19 (3). Moreover, management of patients with chronic dermatological conditions became increasingly challenging during the pandemic, largely due to disruptions in dermatology clinic operations (4). SARS-CoV-2 infection can potentially exert a negative effect on clinical outcomes of pre-existing dermatoses.

Our study provided a novel insight into dermatosis outcomes during the COVID-19 outbreak, revealing that vitiligo, compared to other common dermatoses, exhibited a lower likelihood of aggravation in the COVID-19 era. Vitiligo is a depigmentation cutaneous disorder primarily caused by the destruction of melanocytes, which is mediated by the skin-infiltration of melanocyte-specific CD8+ T cells (5). While cases of *de novo* or flares of vitiligo triggered by the COVID-19 vaccines have been reported (6), they are generally rare and mild. A retrospective study conducted in China identified treatment delays as the most important risk factor for vitiligo progression and recurrence during the pandemic (7). However, during our study period, treatment delays were no longer a predominant concern, given the relaxation of COVID-19 regulations. The inflammatory response triggered by COVID-19 appeared to have a relatively weaker impact on the prognosis of vitiligo compared to other dermatoses.

Psoriasis is a chronic inflammatory skin condition that can be exacerbated by hyperinflammation and cytokine storm associated with COVID-19. Temporary exacerbation of pre-existing psoriasis following COVID-19 booster vaccination (8) and worsening of psoriasis due to COVID-19 (9) have been reported. Our results showed that a higher T_max_ during COVID-19 could elevate the risk of psoriasis aggravation. The underlying mechanism is likely to involve a more intense cytokine storm, relying on further investigation to be explored. Previous studies have revealed that the use of biologics was not associated with an elevated risk of contracting COVID-19 or with a more severe COVID-19 disease course (10, 11). In line with previous reports, our study suggested that biologics treatment did not aggravate the outcomes of pre-existing psoriasis, which may be explained by the suppression of cytokine storm by biologics therapies.

Furthermore, biologics treatment was related to a reduced risk of AIBD aggravation during the COVID-19 outbreak. In our study, seven AIBD patients received biologics treatment and none of them experienced aggravation. However, an association between Rituximab use in AIBD and a higher probability of COVID-19 has recently been reported (12). Dermatologists must carefully weigh the benefits and risks when prescribing biologics in the context of the COVID-19 pandemic.

This observational study provides a snapshot of the clinical outcomes of pre-existing dermatoses during the first phase of the Omicron outbreak in China, following the easing of restrictions. With a substantial sample size, the study stands as the first study to compare the outcomes of different major dermatoses during the COVID-19 pandemic. Data collection occurred through in-person consultations, ensuring effective doctor-patient communication. Our studies had a few limitations. Enrolling a sufficient number of SARS-CoV-2 uninfected patients during the study period was challenging, and only mild COVID-19 cases not requiring hospitalization were included. Many major dermatoses were not covered, particularly hidradenitis suppurativa (only one case was included) and vasculitis/vasculopathy. The flaring of hidradenitis suppurativa upon COVID-19 vaccination and infection is garnering increasing attention (13). Similarly, vasculitis/vasculopathy has been reported to relapse following both COVID-19 infection, especially with earlier variants, and vaccination (14).

## 5 Conclusion

In conclusion, this study reveals a lower propensity for vitiligo to aggravate during the COVID-19 outbreak compared to other dermatoses. Furthermore, a higher body temperature during COVID-19 infection can increase the risk of aggravation for psoriasis. Biologics treatment emerges as a protective factor against the aggravation of AIBD during the COVID-19 outbreak. To assess the long-term effects of the Omicron pandemic on the prognosis of major dermatological diseases, larger worldwide studies are anticipated.

## Data availability statement

The raw data supporting the conclusions of this article will be made available by the authors, without undue reservation.

## Ethics statement

The studies involving humans were approved by the Bioethics Committee of Peking Union Medical College Hospital. The studies were conducted in accordance with the local legislation and institutional requirements. The participants provided their written informed consent to participate in this study.

## Author contributions

J-HL: Conceptualization, Formal analysis, Investigation, Validation, Writing – original draft, Writing – review & editing. S-ZL: Conceptualization, Data curation, Formal analysis, Investigation, Software, Writing – review & editing. S-HW: Conceptualization, Data curation, Investigation, Methodology, Writing – review & editing. JZ: Investigation, Validation, Writing – review & editing. Y-HX: Investigation, Software, Writing – review & editing. Y-GZ: Conceptualization, Funding acquisition, Methodology, Supervision, Validation, Writing – review & editing.
