# Supplementary material for: Factors influencing the outcomes of dermatoses during the COVID-19 outbreak in China: a retrospective study
Source: Front Med (Lausanne). 2024 May 30;11:1417358. doi: 10.3389/fmed.2024.1417358 (PMC11169820; doi:10.3389/fmed.2024.1417358)
Supplement: Supplementary file 1 [file Table_1.docx]

Supplementary Table 1 Comparison between patients with aggravation and non-aggravation outcomes of other dermatoses.

| Eczematous disorders | | | | |
| --- | --- | --- | --- | --- |
| Characteristic | Aggravation (n=57) | Non-aggravation  (n=145) | *P*-value | OR (95% CI) |
| Age, in years | 43.7 (± 15.9) | 43.0 (± 19.7) | 0.824 |  |
| Sex (female) | 29 | 74 | 0.984 | 1.006 (0.545–1.858) |
| Biologics treatment | 0 | 3 | 0.560 | 0 (0–4.623) |
| SARS-CoV-2 infection | 45 | 121 | 0.452 | 0.744 (0.343–1.611) |
| Fever | 42 | 108 | 0.429 | 1.685 (0.457–6.214) |
| T_max_ (>38.6 °C) | 25 | 53 | 0.250 | 1.526 (0.741–3.143) |
| Fever duration (>2 days) | 35 | 79 | 0.190 | 1.835 (0.734–4.589) |
| Disorders of pilosebaceous units | | | | |
| Characteristic | Aggravation (n=18) | Non-aggravation  (n=69) | *P*-value | OR (95% CI) |
| Age, in years | 30.7 (± 11.3) | 25.6 (± 10.9) | 0.081 |  |
| Sex (female) | 11 | 42 | 0.985 | 0.990 (0.342–2.868) |
| SARS-CoV-2 infection | 16 | 58 | 0.609 | 1.517 (0.305–7.553) |
| Fever | 16 | 56 | 0.612 | ∞ (0.078–∞) |
| T_max_ (>38.6 °C) | 9 | 35 | 0.651 | 0.771 (0.250–2.379) |
| Fever duration (>2 days) | 13 | 37 | 0.133 | 2.225(0.564–8.773) |
| Vitiligo | | | | |
| Characteristic | Aggravation (n=11) | Non-aggravation  (n=60) | *P*-value | OR (95% CI) |
| Age, in years | 17.3 (± 13.8) | 27.0 (± 16.0) | 0.063 |  |
| Sex (female) | 7 | 20 | 0.057 | 0.286 (0.075–1.092) |
| SARS-CoV-2 infection | 10 | 52 | 0.378 | 1.538 (0.173–13.695) |
| Fever | 10 | 51 | 0.839 | ∞ (0.010–∞) |
| T_max_ (>38.6 °C) | 7 | 27 | 0.175 | 2.074 (0.482–8.931) |
| Fever duration (>2 days) | 7 | 40 | 0.254 | 0.642 (0.142–2.899) |
| Urticaria | | | | |
| Characteristic | Aggravation (n=7) | Non-aggravation  (n=14) | *P*-value | OR (95% CI) |
| Age, in years | 43.6 (± 13.0) | 40.5 (± 13.9) | 0.631 |  |
| Sex (female) | 4 | 7 | 0.341 | 0.750 (0.121–4.662) |
| SARS-CoV-2 infection | 7 | 12 | 0.533 | ∞ (0.142–∞) |
| Fever | 7 | 10 | 0.386 | ∞ (0.167–∞) |
| T_max_ (>38.6° C) | 3 | 6 | 0.302 | 0.500 (0.070–3.550) |
| Fever duration (>2 days) | 6 | 10 | 0.412 | 0 (0–13.300) |

Abbreviations: OR, odds ratio; CI, confidence interval; T_max_, maximum body temperature.
